# Hospital and Institutional News

**Published:** 1912-08-17

**Authors:** 


### HOSPITAL AND INSTITUTIONAL NEWS.

### "THE ADVANCE OF THE POOR-LAW INFIRMARY."

We publish elsewhere in this issue a most interesting interview with Dr. W. R. Jordan, visiting physician to the Birmingham Union Infirmary, in which he exemplifies, from the progressive development of that institution, the advance of the Poor-Law Infirmary to its present high position as the friendly rival of the best voluntary hospitals of to-day. Dr. Jordan, whose experience of infirmary developments at Birmingham proves him well qualified to speak, has some illuminating points to make with reference to the peculiar value of a visiting staff, and defines within a few terse sentences that elusive quality commonly known as the " institutional atmosphere." He shows what this means, how it is fostered, and the best method of counteracting its influence, besides examining the part which classification has played in infirmary progress. Dr. Jordan also pays a well-deserved tribute to the really great work which the lately retired Matron, Miss Gibson, did for the nursing department during the long period, which began almost with the infirmary, that she was in control. Few readers of this interview will dispute Birmingham's claim to have been a pioneer in that humanising of our public hospitals which may justly be termed the advance of the Poor-Law Infirmary.

### THEFTS AT OPERATIONS.

Only a week or two ago we had to call attention to the desirability on general grounds of limiting visitors at surgical operations to qualified medical men, medical students, and trained nurses. This limitation was made necessary by the frequent applications which certain hospitals receive from members of first aid and other societies. Since our article was published, a more personal reason for care in allowing the presence of visitors has presented itself. A German medical student, Walter Denuth by name, has been gaining access to the London hospitals by presenting himself, the story goes, as a medical man, with the result that three physicians and one surgeon appeared at Westminster Police Court on Tuesday last week to prosecute him for thefts of money and personal belongings. Dr. Henry L. Woodroffe, of the Cancer Hospital, had lost a watch worth £15; Dr. W. J. Gow, of St. Mary's, had lost a pocket-book and some loose cash; Dr. Lyndon Ley, acting registrar at the Chelsea Hospital for Women, had lost a watch and chain worth £35; and Mr. Thomas Perry Legg, surgeon to King's College Hospital, said that his gold sovereign purse and £7 10s. had been stolen. The prisoner's method apparently was to gain an invitation to watch an operation, during which he would excuse himself on some pretext, ransack the surgeon's clothes, and disappear. Mr. Francis, who, by the way, severely criticised the pawnbroker for accepting a sovereign case without inquiry although engraved with the owner's name, sentenced the prisoner to deportation, to succeed nine months' hard labour. It would thus seem that the disturbance at operations caused by movement or " faintness " on the part of visitors whose claim to be present has not been fully investigated may be used for criminal purposes. This practical warning comes pat on the general restrictions we felt compelled to advocate three weeks ago.

### ENGLISH DOCTORS IN GERMAN PRISONS.

The English doctors, who, as we noted last week, were arrested during their holiday trip as yachtsmen near Kiel as supposed spies, were set at liberty on August 8. Immediately on their release the Englishmen went on board their yacht, and Dr. N. Roberts arrived in London on Friday last week. He at once visited Dr. Alan Moore at St. Bartholomew's Hospital, and we understand that he and his friends were not, in spite of the favourable reports concerning their treatment which appeared in the Berlin newspapers, treated after all with such great consideration. It seems, in fact, that they were six hours without food after their arrest, and that after being placed respectively two in one cell and three in another, suffered solitary confinement for at least a couple of days, except for the privilege of meeting at meal times. Dr. Roberts, moreover, is stated to complain of the dirtiness of the cell in which he was placed, no expression of regret for which appears to have accompanied the order for release. The " spy " panic is becoming a perennial nuisance in both Germany and England, regardless, apparently, of the expert military opinion to the effect that nothing worth knowing can be effectively concealed. The science of war is largely, like the other sciences, cosmopolitan in the twentieth century, and doctors and other holiday visitors to the Continent should certainly be allowed to take half a dozen snapshots, which very likely will never survive development, without exciting two nations to the verge of panic. In England, at any rate, even for the purposes of sensational lay journalism, the " arrest of a spy " is becoming a played-out topic of news, and the treatment meted out to Dr. Roberts in this case was certainly quite unwarrantable.

### A RECORD AT IPSWICH MENTAL HOSPITAL.

Perhaps no better test of good administration in a mental hospital can be found than the length of time which the attendants stay. It is thus interesting to note that the Commissioners in Lunacy in their Report on Ipswich Mental Hospital record the fact that 50 per cent. of the attendants show more than five years' service. No doubt such questions as superannuation and allowances all tend towards stability in a mental hospital staff, but it has been proved again and again that the expectation of future rewards is unable to retain attendants and others if not reinforced by considerate treatment as to diet, recreation, and general well-being. In this connection it is interesting to note the experiment for a set diet made in another mental hospital, which we recorded in The Hospital of last week. Such considerations are of the utmost importance to successful administration, and no doubt Dr. E. L. Rowe, the medical superintendent at Ipswich, could furnish more hints than one to explain the satisfactory record of service which the Commissioners noticed. No work is more exacting than work among mental patients, and we are glad to note the steady efforts that are being made generally to respect the strain and to compensate it by adequate rest, change of diet, and opportunities for recreation.

### A HOSPITAL AND WORKING-MEN AT NEWCASTLE.

Whatever may be the general effect of the Insurance Act upon workmen's subscriptions to the voluntary hospitals, we have good authority for stating that the rumour, which found currency in certain London lay newspapers last week, that the Walker Accident Hospital, Newcastle-on-Tyne, has suffered a serious diminution in this, the principal source of its income, is not countenanced officially either at the various works or at the hospital. So strong a tide of working-men's support is revealed in the latest report of the institution for the year ending June 30, that were it suddenly to ebb there could be no doubt or hesitation on the point. Last year's figures speak for themselves. The workmen's quarterly subscriptions and the employers' special subscriptions both showed a substantial increase on the previous year's total, and the annual subscriptions were only less by under £5. The net result is a balance in hand of some £200, which it is proposed to devote towards the expense of building a new and up-to-date operating-room and surgery, the present surgery being used as a waiting-room. It is an object with which the working-men are likely to sympathise, since its aim is to obviate the discomforts of waiting that are inseparable from the smallness of the present accommodation; when, as has lately been the case, over fifty cases require to be dealt with in a morning, the existing surgery and waiting-room have been unduly over-strained. The proposed new scheme, plans for which have been prepared by Mr. E. F. W. Liddle, is now under consideration. This being the position of affairs, Col. R. Saxton-White, the chairman, and Mr. William Bourn, the hon. secretary, can hardly fail to regard the ill-informed rumour as singularly inopportune.

### BUILDING ACTIVITY IN BIRMINGHAM.

It is proposed to extend the Birmingham City Sanatorium at Bordesley Green. Messrs. W. H. Ward and Son have prepared plans which show new women's pavilion, dining and recreation rooms, children's pavilion and day rooms, observation and administrative blocks, with laundry and boiler-house. The cost of the works, including laying out the grounds, is expected to be about £30,000. The Health Committee proposes to establish a dispensary in Broad Street, and Mr. J. G. Dunn has prepared plans for the necessary alterations which will provide for laboratory and examination rooms. The work is estimated to cost about £4,000. It is also proposed to remodel the Lynden End Hospital so as to fit it for use as a sanatorium for consumptives. The Hospital Saturday Committee is erecting at Romsey Hill, about nine miles from Birmingham, a consumptive sanatorium. The advisability of enlarging the Salterly Grange Sanatorium near Cheltenham is also under consideration, and it is proposed to recommend alterations and additions to the West Heath Sanatorium to bring it into line with present day requirements. It would seem, in short, that the "Sanatorium Benefit" is giving more opportunities to architects in Birmingham than to the profession elsewhere.

### A NOVEL EAST END COLLECTION.

During the distress in the East End consequent upon the dock strike, now happily ended, a collection of a somewhat novel nature was carried out in Walthamstow. Some 5,000 appeals were forwarded by the organisers to householders asking for food, etc., with each appeal being enclosed a small coloured card bearing a white cross. Those who were willing to contribute were asked to place the card in the window, and upon the day appointed a cart called for the provisions. A correspondent suggests that a similar method of organisation might be of service for hospital "Pound Days," and that many would thus be reached who now contribute nothing. It is possible, of course, that the *vis inertiæ* of human nature may prevent some otherwise well-disposed persons from paying a personal visit to the hospital, which is the manner in which the "pounds" are so frequently delivered, the givers acting as collectors also. But if this be so, it must also be remembered that the semi-social function which the present system entails is also not the least attractive element in a "Pound Day's" success.

### A NEW REGIUS PROFESSOR OF MEDICINE.

Mr. Ashley Watson Mackintosh, M.A., M.D., has been appointed Regius Professor of Medicine in the University of Aberdeen in the place of Professor David White Finlay, who has resigned. Dr. Mackintosh, who was educated at Aberdeen University, took his M.A. degree with double first-class honours in 1888, his M.B., C.M., with high honours in 1893, and his M.D., also with honours, in 1896. His appointments have included those of physician and lecturer in clinical medicine at Aberdeen Royal Infirmary. He has also been clinical assistant at Great Ormond Street Hospital for Children and at the National Hospital for the Epileptic, Queen Square. Dr. Mackintosh is also the author of several works on cerebral disease.

### STRANGE DEATH OF A MENTAL PATIENT.

A strange death was the occasion for an inquest on a patient at Littlemore Mental Hospital, near Oxford, on Monday. Jessie Brooker, the patient, it appeared from the evidence, was one of 205 patients who were in charge of the head attendant and several nurses in the airing court. On being counted as they returned to the wards Brooker was missing. A search resulted in her being discovered in an acacia tree which was growing in the recreation ground. In spite of every inducement, including the placing of a fire escape in a suitable position against the tree, Brooker refused to come down. In fact she climbed to the top of the tree and then fell head foremost into one of the blankets which were being held out. Her weight and the height of her fall were such as to make the blanket impinge upon the gravel, and the patient's neck was dislocated. She also sustained a fractured skull. A verdict of accidental death was returned, and the opinion was expressed that no blame attached to the Littlemore authorities. Brooker had been a patient since January last.

### FROM GUARDSMAN TO MENTAL SUPERINTENDENT.

It is not often that the same man holds such different posts as the two named in the title of this paragraph, but that he did so is not the least interesting fact about the life of Dr. Thomas Lawes Rogers, who died at Eltham last week at the age of eighty-three. After qualifying in England in 1853, Dr. Rogers gained the M.D. of St. Andrews in 1857 and took the M.R.C.P. in 1860. A year after qualifying he joined the 1st battalion of the Coldstream Guards, obtaining a commission as assistant surgeon, and served with them through the Crimean War, for which he gained the medal. He retired from the Army at the early age of twenty-nine and became Medical Superintendent of the Rainhill County Mental Hospital in Lancashire, to which, indeed, he had acted as assistant superintendent for one year before joining the colours. He retired in 1888, only, however, to continue his hospital work on various committees, becoming a member of the committee of management of the "Dreadnought" Seamen's Hospital in 1893. Here he found additional outlet for his energies in the process of converting the old building into a modern hospital, and in promoting the then new School of Tropical Medicine at the Docks. He also was a member of the committee, formed by Mr. Joseph Chamberlain and presided over by Mr. Percival Alleyne Nairne at the Colonial Office, which drew up the syllabus. In the "Seamen's" his interest never flagged, and he was appointed a vice-president in 1909. The Workshop for the Blind in Kent and the Charity Organisation Society also knew him. Being possessed of considerable private means he retired to Eltham, where he purchased "East Bank" and resided till his death. So varied a career is interesting to all of us, and perhaps is only possible when the chance of war offers unforeseen opportunities to those who seem steadily planted on the medical or institutional pathways of life.

### MEDICAL MEN AS PRISON GOVERNORS.

Mr. Robert Nesmith Paton, L.R.C.P., late Medical Officer to his Majesty's Prison, Wormwood Scrubs, has been appointed by the Home Office Governor of Holloway Prison in succession to Dr. James Scott, M.B., who has retired through ill-health. Both gentlemen, it is interesting to observe, were educated at Edinburgh, where Dr. Scott graduated in 1877, and where Dr. Paton gained his L.R.C.P. and S. in 1878. There is thus seen a tendency to recognise the suitability of medical men for the responsible and highly difficult work of prison governor. Such a post is easy to no type of man, but the doctor, who is in touch with modern psychiatry upon the Continent, and who knows how many "criminals" prove on scientific examination to be diseased rather than evil persons, should be able to combine knowledge and sympathy in his work as governor such as no other profession can do. And yet hard things have been said of prison doctors, chiefly because they have been engaged not as whole-time officials, but as holding other appointments, or as devoting their chief energies to private practice. When educated men who are also trained observers, especially if they happen to possess from their reputation the public ear, as is sometimes the case, get into prison, a severe indictment of the chaplains and the doctors is usually the result. We quote here a classic example: "The officials who should not be allowed to hold any employment outside the prison, or to have any private practice, are the prison doctors. At present the prison doctors have usually, if not always, a large private practice and hold appointments in other institutions. The consequence is that the health of the prisoners is entirely neglected and the sanitary condition of the prison entirely overlooked." Omitting some harsh criticisms on the prison doctors which then follow, the writer continued: "If prison doctors were prohibited from private practice they would be compelled to take some interest in the health and sanitary condition of the people under their charge." These words were written twelve years ago. A still more promising remedy has been adopted by making the prison doctor not merely a whole-time official, but the chief official in the prison.

### ORGANIST AND HOSPITAL GOVERNOR.

Mr. T. R. Ryder, B.A., who for the past twenty-four years has acted as hon. organist and choirmaster to the Leicester Royal Infirmary Chapel, has recently resigned the position, and, to mark their appreciation of his long service, the Governors at the half-yearly meeting unanimously elected Mr. Ryder a Life Governor of the institution.

### DOCTORS' FEES AMONG THE ESKIMO.

A very interesting series of letters has been published in summary by the *Times* from Stefansson, one of the Anglo-American expedition to the hitherto believed uninhabited regions of Northern Canada. In one of them, dated from Shingle Point, he refers to the medical practices that he found among the Eskimo and other tribes. "There are two ways," he is quoted as saying, "of treating diseases or injuries among the Kogmolliks (Herschel Island to Parry). These are : (1) Blood-letting (apparently referred to by some writers as 'counter irritation') and (2) magic. Whether the latter is exorcistic in purpose I have not yet learnt. The gashes for blood-letting are one and a half or two inches long and skin-deep. They are over the real or fancied seat of the disease, or near it, and are cut by the man himself, or anyone who is present when the patient decides he wants the cut made; there is, of course, no fee, though a second person does the work. Certain men in the community are known as doctors. The statements as to how they become doctors are inconsistent. Their treatment is by songs and dances, with sometimes a sleight-of-hand trick or two, and neither the invalid nor the audience takes any active part. The details are somewhat complicated, and I have seen no performance so far. After the performance the doctor is paid—in the old days the fee went as high as two to three whaling uniaks (big skin boats). If the invalid is poor everybody gives the doctor something—a fox-skin, spear, bag of oil, or other things of value. If the man treated dies within the year his wife or children get the fee back—under other conditions the fee is retained."

### THE FIRST APPLICANT FOR A BENEFIT REFUSED

Sanatorium benefit, of which so much has been heard of late, was refused last Saturday to a clerk in the Dorset County Council offices, who made an application on behalf of his wife. The Dorset County Insurance Committee, however, resolved, in view of the uncertainty as to how much money would be required for insured persons, not to assist for the time being any insured person's dependant. This treatment of the first applicant will hardly encourage others to apply.

### PAYING THE COST OF SANATORIUM BENEFIT.

An order was issued by the National Health Insurance Commissioners last Monday stating that to defray the expenses of sanatorium benefit during the period between July 15, 1912, and January 12, 1913, deductions will be made from the sums credited to deposit contributors and to approved societies respectively. The Commissioners will determine the amounts to be deducted according to their estimate of the sum needed for meeting the cost of providing sanatorium benefit during the period, but such amounts will not exceed one penny for every four contributions paid in respect of the deposit contributor, and one farthing for every contribution credited to an approved society. All sums thus deducted are to be transferred to the proper Insurance Committees. No sums will be deducted from those deposit contributors who are not entitled to sanatorium benefit.

### SIR THOMAS BOOR CROSBY AT AIX.

The Lord Mayor of London meets with honours everywhere. He had hardly arrived at Aix-les-Bains when a banquet was given in his honour by the Aix Medical Society. Sir Thomas Boor Crosby was welcomed by Dr. Goddard, the President of the Society, and by the Mayor of the town. Among the medical men of different nationalities present were three of Sir Thomas's compatriots, Dr. Hunter, Dr. Tod, and Dr. McCann, of London.

### THE LORD MAYOR AND ST. THOMAS'S HOSPITAL.

One of the acts which will mark the present Lord Mayor's last month of office will be connected with hospitals, since Sir Thomas Crosby is to take the chair at the Old Students' dinner of St. Thomas's Hospital on October 1. As soon as Sir Thomas Crosby's election as Chief Magistrate was announced it was prophesied that his year of office would be remarkable not merely from the fact that he was the first medical man to become Lord Mayor of the Capital, but also because the cause of hospitals and medicine would inevitably be better appreciated at the Mansion House than ever before. That forecast has been well fulfilled. Few weeks have passed when we have been unable to chronicle some act of the Lord Mayor's which has forwarded the cause of medical science or the voluntary hospital system; and it requires no effort of memory to recall the St. Bartholomew's Hospital dinner held at the Mansion House, his continual plea to the profession to enter more readily into the public life of the nation, or his personal effort on behalf of Hospital Sunday, when he wrote an article urging its claims in our Hospital Sunday Special Number. Towards the end of these activities it is, therefore, graceful and fitting that he should return to his own hospital's affairs and preside at its Old Students' dinner, which on that account alone should be a remarkable occasion. It will remind many also in a personal way how much the profession owes to the City's Chief Magistrate.

### COMING ADVANCE IN LAUNDRY PRICES.

A threatened increase in prices for all laundry work in the London area is expected and will probably be in force by October. Hospitals with their own laundries will be in a position to take this change cheerfully, but for those not self-supporting in this respect much will depend upon the date of expiration of their contracts. The Launderers' Association has, of course, many excuses for the decided change; at the beginning of last month, the Association states, policies for laundry hands under the Workmen's Compensation Act were increased in cost by 25 per cent. Coke is quoted as 20 per cent. more than it was three months ago; yellow soap, the calico stretched over the ironing boards, and the Insurance Act are all to blame. Institutional housekeepers will learn with interest that the Association proposes to recover its losses by abolishing the "contract work," which takes the form of new customers insisting on their servants' washing being done at a flat rate of between 1s. and 1s. 6d. a head. Laundry proprietors have found that the servants gain by this system, and desire to return to the policy of charging the servant three-quarters of the tariff paid by the mistress. If this be not accomplished an increase of about 1d. in the 1s. is likely to follow.

### A HERO OF THE CHOLERA EPIDEMIC.

The death of Dr. James Scott Sequeira removes a landmark in the rise and development of medical officers of health. Born in 1828, he came of a Portuguese family connected long and honourably with medicine. His great-grandfather, a native of Lisbon, died in 1816 the oldest Licentiate of the Royal College of Physicians. He himself was born in Whitechapel and practised under his father, and after a visit to Australia he returned to his former part of London and eventually became and held for thirty-four years the post of Medical Officer to the Whitechapel Union. In those days the freedom allowed to medical officers was small, and an epidemic was invariaby accompanied by serious loss of life, as those who understand the motives and work of Edwin Chadwick will need no reminding. If Sir Edwin's name has a place in history as that of a pioneer of municipal sanitation, and a preacher of that now so sincerely respected "gospel of drains," Dr. Sequeira will also be remembered as one of the first medical officers with a keen sense of the general ignorance and an active desire to meet the emergencies which were created by its prevalence. Some fifteen years ago Dr. Sequeira retired from practice full of anecdotes of the cholera epidemic, which alone, it must never be forgotten, was sufficient to secure public attention to the want of drainage and hideous insanitation of London.

### THE RETIREMENT OF SIR PATRICK MANSON.

On his retirement from the post of Medical Adviser to the Colonial Office Sir Patrick Manson (who recently was decorated G.C.M.G.) was, it will be remembered, elected to the honorary medical staff of the Seamen's Hospital Society, with duties as physician at the Albert Dock Hospital, in 1892. It was on his initiative in 1898 that Mr. Joseph Chamberlain invited the committee of management to establish the London School of Tropical Medicine. Sir Patrick's resignation has now been accepted by the committee of the hospital, and he has been duly elected consulting physician to the Society. As all will be glad to know, Sir Patrick Manson retains his appointment as senior teacher in the London School of Tropical Medicine, but he will be absent from the School for some time as he is shortly sailing for Ceylon, where his son-in-law, Dr. Philip Bahr, is now engaged as one of the travelling scholars of the School in carrying out an investigation into the origin and causation of sprue.

### A LIBEL ON HOSPITAL DISPENSERS.

Mr. E. Saville Peck, M.A., dealing with the training of apprentices in pharmacy at the British Pharmaceutical Conference (Practice Section) last month, referred to the unequal supply of apprentices, and attributed the deficiency to the comparatively long hours, the so-called shop drudgery, and the inadequacy of the eventual remuneration. An otherwise admirable paper was spoilt by a reference to such places where, in the author's opinion, an insufficient training was obtained, amongst which he included "the hospital dispensary, where a sufficient number of hours daily or weekly is spent to enable the pupil to make the necessary declaration for entering for the qualifying examination." There are, we are sure, very few hospitals or dispensaries which allow their pharmacists to instruct pupils without having the necessary equipment for an honest training, and there is nowhere else in the kingdom where more pure pharmacy is practised than in these institutions. It is to be regretted that the Chairman, Mr. Edmund White, F.I.C., did not correct Mr. Peck upon this matter, which, as a past pharmacist of St. Thomas's Hospital, he would be well able to do.

### THE EFFICIENCY OF REGISTRATION.

A good service has been done to the progress of vital statistics by the publication of the Report which was undertaken by a Special Committee of the Royal Statistical Society appointed to inquire into the systems adopted in different countries for the registration of births (including still-births) and deaths with reference to infantile mortality. The Empire, Europe, and such distant places as Uruguay have been sought for information, and, the Committee states, the efficiency of registration is found to be very fairly complete, at least in the more settled countries. The Committee's weighty remarks on vital statistics deserve quoting: "Vital statistics may, in effect, be regarded as taking the place of laboratory experiments in social physiology and pathology," and their principal function is explained as the examination of the causes of preventible wastage of life. The Committee recommends a greater uniformity in the practice of calculating fertility and mortality rates. Its conclusions comprise that still-births should be tabulated separately. That, generally, the infantile mortality rate should be calculated from the births of children born alive but registered dead, and from the number of deaths during the first year of life of children born alive. The Report is divided into four sections: A history of registration, its general practice, still-births, and statistical methods. Such a collection of data and the comparisons which they afford should prove invaluable to the new science, the importance of which, thanks largely to the splendid efforts of Professor Karl Pearson in specific fields, is being gradually appreciated.

### TWO IMPROVEMENTS AT A LIVERPOOL HOSPITAL.

Two important improvements are being made at the Royal Southern Hospital, Liverpool, and it is an interesting fact that both are being carried out in the form of memorials. For some years past additional accommodation for infectious and doubtful cases has been much needed, but no room was available for the purpose until the work of the old out-patient department was transferred to the new building opposite to the hospital. The space thus set free is now being used for two observation wards, containing respectively four beds for men and three for women, and two single-bedded isolation wards for suspicious cases before admission, or infectious cases pending their removal to isolation hospitals. These wards should increase the efficiency of the hospital by enabling patients to remain for a time sufficient for full diagnosis, by minimising the transfer of cases to workhouse hospitals, and by reducing the risk of infection arising in the wards. The contract for the work amounts to £1,370, and Mrs. W. G. Killick is to be congratulated on choosing so excellent a scheme in memory of her late husband, who for thirty-five years served on the committee and for fourteen years presided over the weekly board. Mr. and Mrs. J. Watson Hughes have likewise provided a memorial to their little daughter by a generous contribution of £500. The money is to be spent in erecting a sun balcony, so necessary an adjunct to every children's ward, but one which hitherto the children in the Royal Southern Hospital have not possessed. The sanitary arrangements of the ward will also be brought up to date, and the kitchen refloored and modernised. The architect for both improvements is Mr. T. W. Haigh, of 3 Exchange Street East, and the contractor Mr. Arthur Lloyd, of Walton.

### AN APPEAL AND A QUESTION.

That very old and celebrated institution, the Royal Maternity Charity of London, has lately received less support than its distinguished record and its excellent past work entitle it to obtain from the public. To remedy a state of affairs which has become really critical, an appeal has been issued by the Princess Christian, president of the hospital, and an influential committee, which includes also Evelyn Lady Stanhope, Lady Iddesleigh, Lord Avebury (treasurer), Mr. R. Inigo Tasker (chairman), Mr. John Whittington (vice-chairman), and the members of the medical staff. The foundation of this institution dates back to the days of George II., since which time it has succoured 564,000 poor married women. The Maternity Charity sends not only skilled medical and nursing assistance to the homes of its patients, but also, since these are of the very poorest classes, provides what is often almost more important still, material comforts to alleviate the suffering of those who have to face the ordeal of parturition in a destitute condition. It gives us great pleasure to afford such publicity as we may to the needs of this institution, and we trust that an adequate response may be aroused. But we note that subscriptions fell last year to the low figure of £365, and we think the management might well inquire how it is there has been such a falling off. Notwithstanding Budgets and Insurance Acts, we think this figure argues remissness somewhere or other. Can the Secretary, Major Killick, throw any light on the point?

### THE WORKERS' FUND AT CAMBRIDGE.

During the year 1910 we had the pleasure of recording the birth of this new organisation, which had been formed for obtaining regular and systematic contributions by workers from workers of all classes to assist in the maintenance of Addenbrooke's Hospital. It was assumed that the bulk of the funds to be collected would be derived from the voluntary weekly contributions of the workers for whose benefit Addenbrooke's and certain other institutions chiefly exist. There are similar organisations, it is true, in a number of towns and cities in other counties of the North and Midlands, but this new organisation in Cambridge differs from them in that it does not confine its contributions to workers at factories, but welcomes contributions from clerks, assistants in both wholesale and retail shops, porters, domestic servants; in fact, from almost every class of worker. One of the rules is that seven-tenths of the net amount collected each year shall be devoted to the exclusive use and benefit of Addenbrooke's Hospital, the remaining three-tenths being retained by the Fund to assist any of its members who may require the benefits of the convalescent homes in connection with it. During its initial year the progress made by this new organisation had given great satisfaction. During the second year of its existence the Fund had to carry on its labours under great difficulties, some contributed by the Legislature, and as a result a number of contributors have been lost. Still, the firms whose employees are supporting this organisation number 111, an increase of five, as compared with that of 1910-11. At the recent annual meeting Mr. D. J. Dyball, chairman of the Fund, presided, and among others present were Mr. E. J. Papworth, hon. secretary, Mr. F. Cant, hon. treasurer, and Miss Montgomery, matron, Addenbrooke's Hospital. The amount collected, including interest, was, less expenses, £610. The hon. secretary, Mr. E. J. Papworth, was given an honorarium of £10 as a slight mark of the committee's appreciation. We congratulate the organisers of the Cambridge and District Workers' Hospital Fund upon the signal success which they have achieved in so short a time.

### THE CLEANING SEASON.

This August, as usual, the cleaners are busy in the London hospitals, full advantage being taken of the holiday season for a general overhauling. The out-patient departments of the Westminster, Royal Free, and New Hospital for Women in Euston Road are now undergoing cleaning and repairs, and, in the public interest, it may be well to give the dates of their reopening. The out-patient department of the Westminster Hospital will reopen on September 12, and those of the Royal Free and the New on September 2. One hospital which has been entirely closed is the Skin Hospital in Stamford Street, Blackfriars; it will, however, reopen its doors on the 29th inst., while the Children's Hospital on Paddington Green reopens at the end of the present week.

### THIS WEEK'S DRUG MARKET.

A quiet tone still prevails in the Drug market, but this is to be expected during the present season. Several drugs, notably quinine and opium, are being watched with interest by prospective buyers. There has been a good demand for quinine in view of the possibility of an advance in price. The position of opium is rather uncertain, but the general impression is that before long a not inconsiderable reduction in value will take place. Menthol is rather dearer, as also is ergot of rye. Following on the advance in price of citric acid the quotations for iron and quinine citrate, iron and ammonium citrate, potassium citrate, and sodium citrate are slightly higher.

### TO OUR READERS.

Contributions are specially invited from any of our readers to these columns. They should deal with topical subjects and news. They must be authenticated for the information of the Editor only. The minimum payment if published is 5s. There is no hard-and-fast rule as to space, but notes of about twenty lines in length are preferred.

